# Ancient DNA Analysis of the Oldest Canid Species from the Siberian Arctic and Genetic Contribution to the Domestic Dog

**DOI:** 10.1371/journal.pone.0125759

**Published:** 2015-05-27

**Authors:** Esther J. Lee, D. Andrew Merriwether, Alexei K. Kasparov, Pavel A. Nikolskiy, Marina V. Sotnikova, Elena Yu Pavlova, Vladimir V. Pitulko

**Affiliations:** 1 Department of Anthropology, Binghamton University, Binghamton, NY, United States of America; 2 Department of Sociology, Anthropology, and Social Work, Texas Tech University, Lubbock, TX, United States of America; 3 Institute for the History of Material Culture, Russian Academy of Sciences, St. Petersburg, Russia; 4 Geological Institute, Russian Academy of Sciences, Moscow, Russia; 5 Arctic and Antarctic Research Institute, St. Petersburg, Russia; University of Florence, ITALY

## Abstract

Modern Arctic Siberia provides a wealth of resources for archaeological, geological, and paleontological research to investigate the population dynamics of faunal communities from the Pleistocene, particularly as the faunal material coming from permafrost has proven suitable for genetic studies. In order to examine the history of the Canid species in the Siberian Arctic, we carried out genetic analysis of fourteen canid remains from various sites, including the well-documented Upper Paleolithic Yana RHS and Early Holocene Zhokhov Island sites. Estimated age of samples range from as recent as 1,700 years before present (YBP) to at least 360,000 YBP for the remains of the extinct wolf, *Canis* cf. *variabilis*. In order to examine the genetic affinities of ancient Siberian canids species to the domestic dog and modern wolves, we obtained mitochondrial DNA control region sequences and compared them to published ancient and modern canid sequences. The older canid specimens illustrate affinities with pre-domestic dog/wolf lineages while others appear in the major phylogenetic clades of domestic dogs. Our results suggest a European origin of domestic dog may not be conclusive and illustrates an emerging complexity of genetic contribution of regional wolf breeds to the modern *Canis* gene pool.

## Introduction

It is widely accepted that the domestic dog (*Canis lupus familiaris*) descended from the gray wolf (*Canis lupus*), but the process of domestication as well as geographical origin and approximate date of first domestication is still debated [1,2,3]. Genetic studies of modern dog and wolf populations have shown divergent views, from a single origin in East/South Asia [4,5] or the Near East [6] to multiple areas of domestication and/or hybridization with regional wolf breeds [6,7]. Furthermore, the possibility of admixture with other canid species has also been previously suggested [8,9]. On the other hand, recent mitochondrial genome analysis of ancient canids has suggested a European origin of domestic dogs [10]. Archaeological evidence is not always straightforward for the morphological identification of domestic dogs, especially as the earliest dogs were essentially the same size as wolves [11,12,13], but advanced morphometric analyses have improved the efforts [14,15,16]. The oldest archaeological evidence of domestic dog has been identified in western Europe and the Near East, dating to at least 14,000 cal BC [17,18]. Some have argued that domesticated dogs were present prior to the Last Glacial Maximum, but this is currently disputed [13,19,20,21,22].

Archaeological and paleontological research conducted in the Arctic Siberia within past couple of decades have yielded a large amount of bone material suitable for genetic studies, as they mostly come from permafrost deposits that are common in the area. Many ancient DNA studies have focused on extinct Pleistocene or wild species that occupied Siberia [23,24,25,26], but here we focus on the oldest domesticated species *Canis*. Different Canidae species, such as the arctic fox and wolf, were among the Pleistocene arctic fauna that continued into the present [27,28]. Within the region, studies have claimed the presence of dogs in the Russian Plain and Kamchatka by 13,000 cal BC [29,30]. A recent study has suggested the presence of a domestic dog in southern Siberia dated to ca. 33,300 cal BC, which predates the oldest evidence from western Europe and the Near East [22]. However, the Siberian canid remain was morphologically most similar to dogs from Greenland and unlike ancient and modern wolves and putative dogs from central Russia [22]. Sablin and Khlopachev [29] have argued that the presence of Pleistocene dog in the central Russian plain at Eliseevichi I dated to 13,000–17,000 cal BC was the result of domestication *in situ* from local northern wolves. Therefore, the possibility that Late Pleistocene/Early Holocene wolves may have contributed to the regional dog breed remains open.

**Fig 1:**
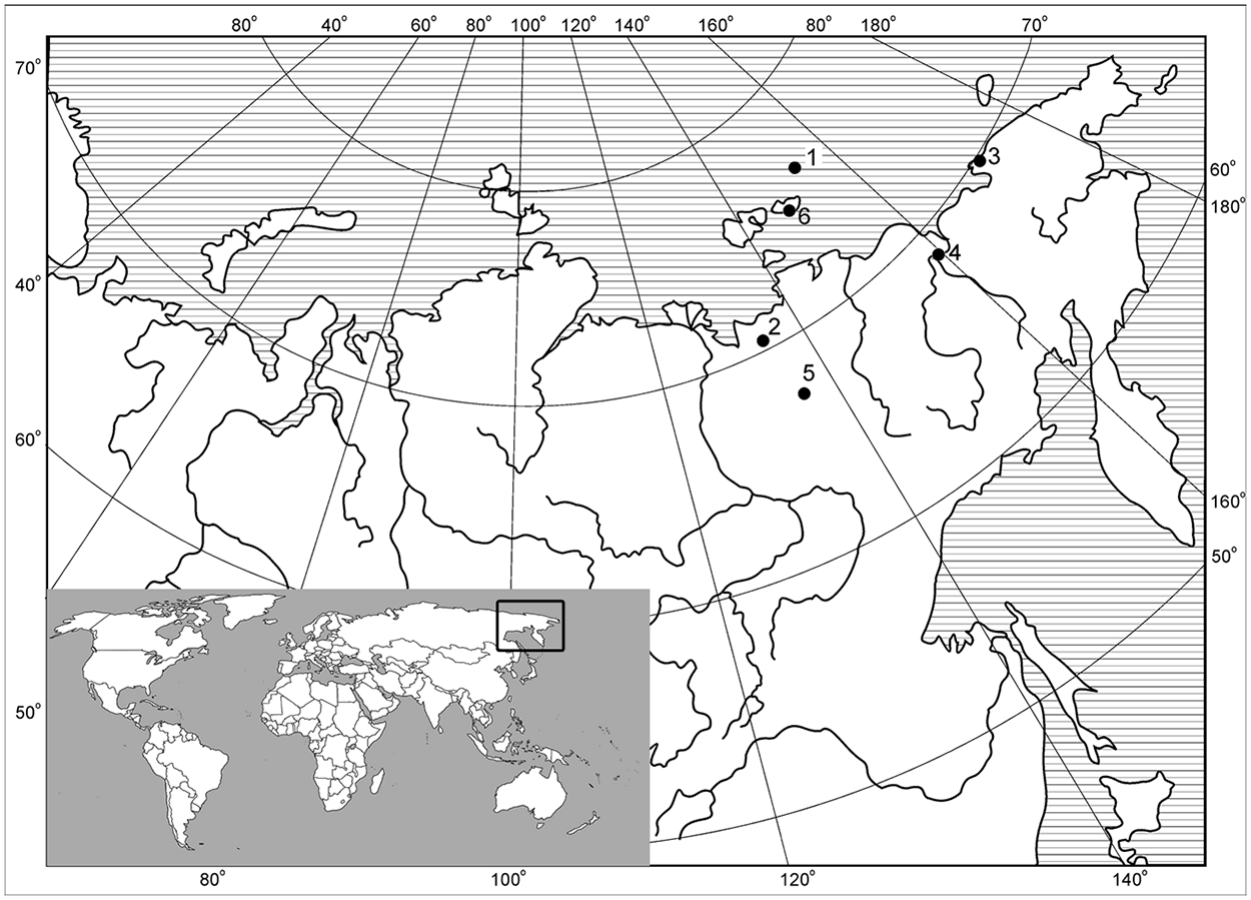
Location of the sites studied. Corresponding numbers and information are provided in Table 1.

We examined thirteen prehistoric canid remains and one contemporary wolf sample from the Siberian Arctic: Ulakhan-Sullar, Duvanny Yar, Yana RHS, Zhokhov Island, and Aachim Lighthouse (Fig 1 and Table 1). The oldest specimens come from the exposures of Quaternary deposits (locality #1, *Canis* cf. *variabilis*) and from Duvanny Yar exposure (locality #2), Details of each site are provided in the next section. Mitochondrial DNA (mtDNA) analysis of the canid specimens was carried out to infer the phylogenetic relationships of these ancient canids with modern canid species, with particular focus on the *Canis* cf. *variabilis* specimen from Ulakhan-Sullar that may provide clues to the origin of domestic dogs.

**Table 1:** Description of the canid specimens analyzed in this study.

ID	Location	Morphological Classification	Age (YBP)^a^	Nr. on the map^b^
S809	Ulakhan-Sullar, Adycha River	*Canis* cf. *variabilis*	360,000–400,000	5
S503	DuvanyYar, Lower Kolyma River	*Canis lupus*	>47,000	4
S504	DuvanyYar, Lower Kolyma River	*Canis lupus*	>47,000	4
S501	Yana RHS, Lower Yana River	*Canis lupus*	28,520 ± 240	2
S601	Yana RHS, Lower Yana River	*Canis lupus*	27,840 ± 220	2
S805	Yana RHS, Lower Yana River	*Canis lupus*	na	2
S806	Yana RHS, Lower Yana River	*Canis lupus*	na	2
S502	Aachim, East Siberian Sea Coast	*Canis sp*.	1,740 ± 40	3
S603	Aachim, East Siberian Sea Coast	*Canis sp*.	1,760 ± 40	3
S602	Zhokhov Island, New Siberian Islands	*Canis sp*.	8,710 ± 50	1
S902	Zhokhov Island, New Siberian Islands	*Canis sp*.	na	1
S903	Zhokhov Island, New Siberian Islands	*Canis sp*.	na	1
S904	Zhokhov Island, New Siberian Islands	*Canis sp*.	na	1
S905	New Siberia Islands	*Canis lupus*	Contemporary	6

Information of the specimens analyzed in this study, including the location, morphological classification, and estimated age.

^a^Age of specimens are based on ^14^C AMS dates (see Supplemental Tables for further details).

^b^Refer to the map at Fig 1 for the site location.

## Materials and Methods

### Sample Information

Our data set consists of five groups of fossils collected in several areas of Arctic Siberia, that cover the area from Zhokhov Island to middle Yana River in latitudinal direction (from 76° 06’ N to 67° 50’ N, or a distance of almost 1000 km) and from low Yana River to Aachim Peninsula, Western Chukotka in the longitude one (from 135° 25’ E to 173° 30’ E, or a distance of 1500 km). The contemporary wolf specimen was collected as a reference sample from the New Siberian Islands [31].

Specimens analyzed in this study have been collected during field excavations (1999–2007) that were conducted under the permits issued by the government agency to Vladimir V. Pitulko, Senior Research Scientist of the Institute for the History of Material Culture, RAS (Zhokhov-2000 project leader): 1) 1999: Credential Letter №123 (form 2—survey), issued by the Field Committee on May 7, 1999, for survey and excavations in Western Chukotka, Pevek district; 2) 2000: Credential Letter №307 (form 2—survey), issued by the Field Committee on May 29, 2000, for survey in New Siberian islands and excavations in Zhokhov island; 3) 2001: Credential Letter №381 (form 1—excavations), issued by the Field Committee on June 8, 2001, for survey in New Siberian islands, excavations in Zhokhov island, and survey in Northern Yana Indighirka lowland; 4) 2002: Credential Letter №638 (form 1—excavations), issued by the Field Committee on June 28, 2002; 5) 2003: Credential Letter №525 (form 1—excavations), issued by the Field Committee on June 16, 2003, for excavations in Zhokhov island, survey in Northern Yana Indighirka lowland, and excavations in Yana site; 6) 2004: Credential Letter №87 (form 4—salvage excavations), issued by the Field Committee on April 16, 2004, for excavations in Zhokhov island and excavations in Yana site; 7) 2005: Credential Letter №506 (form 4—salvage excavations), issued by the Field Committee on June 10, 2005, for excavations in Zhokhov island and excavations in Yana site; 8) 2006: Credential Letter №308 (form 4—salvage excavations), issued by the Field Committee on May 26, 2006, for excavations in Yana site; 9) 2007: Credential Letter №99 and 100 (form 1, excavations, and form 2, survey), issued by the Field Committee on April 13, 2007, excavations in Yana site and survey in Northern Yana Indighirka lowland. All information can be verified through the Field Committee, Institute of Archaeology, Russian Academy of Sciences, at opiiaran@yandex.ru.

### Location and Site Information

#### Ulakhan-Sullar

The mandible of *Canis* cf. *variabilis* (S809) was obtained from Layer 2 dated to 360,000 ± 17,000 years ago based on ESR measurements of bivalve shells found in the same layer [32]. The site is a 65 m-high and 1.2 km-long bluff located at the right bank of low Adycha River (Yana River basin) at 67°41’N, 135°44’E. The species *Canis* cf. *variabilis* was characterized by Teilhard de Chardin and Pei from specimens of small Middle Pleistocene wolves in Asia [33]. Often grouped together with *Canis mosbachensis* in Europe referred to as the *C*. *mosbachensis-variabilis* group, the species shows morphological features including a relatively small body size, elongated and slender snout, and a relatively long metastyle P4.

#### Duvanny Yar

Duvanny Yar is a key section of Upper Pleistocene deposits of Western Beringia, situated on the right bank of the lower Kolyma River in Northeastern Yakutia (68°38’N; 159°03’E) [34,35]. Four stratigraphic units have been characterized at the site, especially Unit III dated to between 45,000 and 13,500 ^14^C years before present (YBP) and has revealed numerous remains of mammals and plant remains [36,37,38,39]. Two canid remains were recovered from the lowermost portion of Unit III, for which radiocarbon dates suggest a date of at least 47,000 ^14^C YBP (S1 Table).

#### Yana RHS

Four specimens of *Canis lupus* were obtained from the site dated to 28,500–27,000 ^14^C YBP [40,41,42]. Located in the low Yana River, the Yana RHS site has yielded rich layers of cultural artifacts and faunal remains, which have been described in detail in previous studies [41,42,43]. Three of four samples have individual ^14^C AMS date (S2 Table), and all together they have a firm age estimation of around 28,000 radiocarbon years BP. Excavations of Yana RHS yielded a number of possible wolf remains, with heavily worn teeth.

#### Zhokhov

The Zhokhov site refers to the Zhokhov Island situated beneath 76°N latitude and belongs to the New Siberian island chain, which constitutes the natural boundary between the Laptev and the East Siberian Seas. Dated to 7,800–8,000 YBP, this is one of the northern most archaeological sites in the Arctic that harbors abundant artifacts and faunal remains, including numerous microprismatic cores and microblades, pieces of hunting equipment, and wooden artifacts [44,45,46]. Canid remains were found among a large artifact assemblage that included faunal remains of polar bear and reindeer, approximating 150 identifiable bones of at least nine animals. Four canid specimens (S602, S902, S904, S903) were included for this study (S3 Table).

#### Aachim Lighthouse

The site is located in the northern part of Aachim Peninsula on the East Siberian Sea coast [47]. Two canid mandibles from this site (S4 Table) are dated to 1,760 ± 40 BP (Beta-231449) and 1,740 ± 40 (Beta-231444), and based on the potential marine reservoir effect the appropriate age accepted would be around 1,700 YBP or a little younger [46]. Despite the age, these specimens predate recent contact, therefore would represent indigenous canid breeds. Apart from the two canid remains recovered that we analyze in this study, the site includes numerous sea mammal remains as well as stone flake and artifacts [47].

### Sample Preparation

All remains except for specimens collected at Aachim remained in permafrost conditions shortly after the deposition event until excavation. Specimens were selected to ensure separate animals (skulls or skull fragments) in order to avoid sampling duplication. Teeth were obtained from cranial and mandibular remains. Remains for a reference material was obtained from a wolf that died of natural causes in New Siberia Island in 2003.

### Experimental Methods

All experimental procedures for DNA analysis were carried out in a dedicated sterile facility for ancient DNA research, which include separate rooms dedicated to drilling, extracting, and preparing samples for PCR. The facility is equipped with high efficiency particulate (HEPA) filtered air, has UV lights over all surfaces, and airflow between the rooms is limited by magnetic interlocks on the doors and by maintaining a serial positive pressure. General practices include the use of disposable garments and gloves, as well as sterilizing exposed surfaces with bleach before each procedure.

Claws and teeth were obtained for DNA extraction. First, specimens were decontaminated with bleach before drilling to produce fine bone powder ranging from 0.45g to 5.45g. Samples were drilled and extracted in groups of five, plus one negative control. Detailed extraction procedures have previously been described [48]. Briefly, samples were first decalcification in EDTA, followed by incubation with proteinase K, then purified using a modified silica-based spin-column protocol followed by centrifugation.

PCR was carried out for the mitochondrial DNA control region using three overlapping primers spanning nucleotide positions (np) 15424 to 15837 (413 basepairs). We used two different primers for the first segment between np 15424 and 15580 as noted in S5 Table. Amplification was carried out multiple times to verify the authenticity. Multiple extracts were used when available and consistent sequences from multiple amplifications were determined reliable. Successfully amplified PCR products were verified by agarose gel electrophoresis and amplicons were purified on a 96-well Multiscreen HTS plate (Millipore, Billerica, MA) to remove single strand DNA and primers. Amplicons were prepared for sequencing using the BigDye Terminator v.3.1 (Applied Biosystems, Foster City, CA). After standard alcohol precipitation, samples were directly sequenced on an ABI PRISM 377XL DNA Sequencer (Applied Biosystems, Foster City, CA).

Sequences were compiled for ancient DNA canid data as well as a representative spectrum of modern dog and wolf samples from the literature and GenBank [4,10,49,50,51,52,53,54,55,56,57,58,59,60,61]. Sequences were aligned using ClustalX [62] and MEGA 5.2.2 [63] and we incorporated our data in three separate median-joining networks that comprised of: 1) ancient DNA canid sequences (N = 33), 2) modern dog sequences (N = 290), and 3) modern wolf sequences (N = 101). Median-joining networks were calculated in Network 4.6 [64].

## Results and Discussion

We successfully obtained endogenous mtDNA control region sequences for all fourteen canid samples and identified nine haplotypes. The two Aachim Lighthouse specimens shared one haplotype and the four remains from Zhokhov Island shared another haplotype. Among the four Yana RHS canid specimens, two shared the same haplotype. Only the Aachim and Zhokhov haplotypes matched other published canid sequences while sequences from Yana RHS, Ulakhan-Sullar, and Duvanny Yar were unique. Sequences can be found under GenBank accession numbers KJ909851-KJ909864.

**Fig 2:**
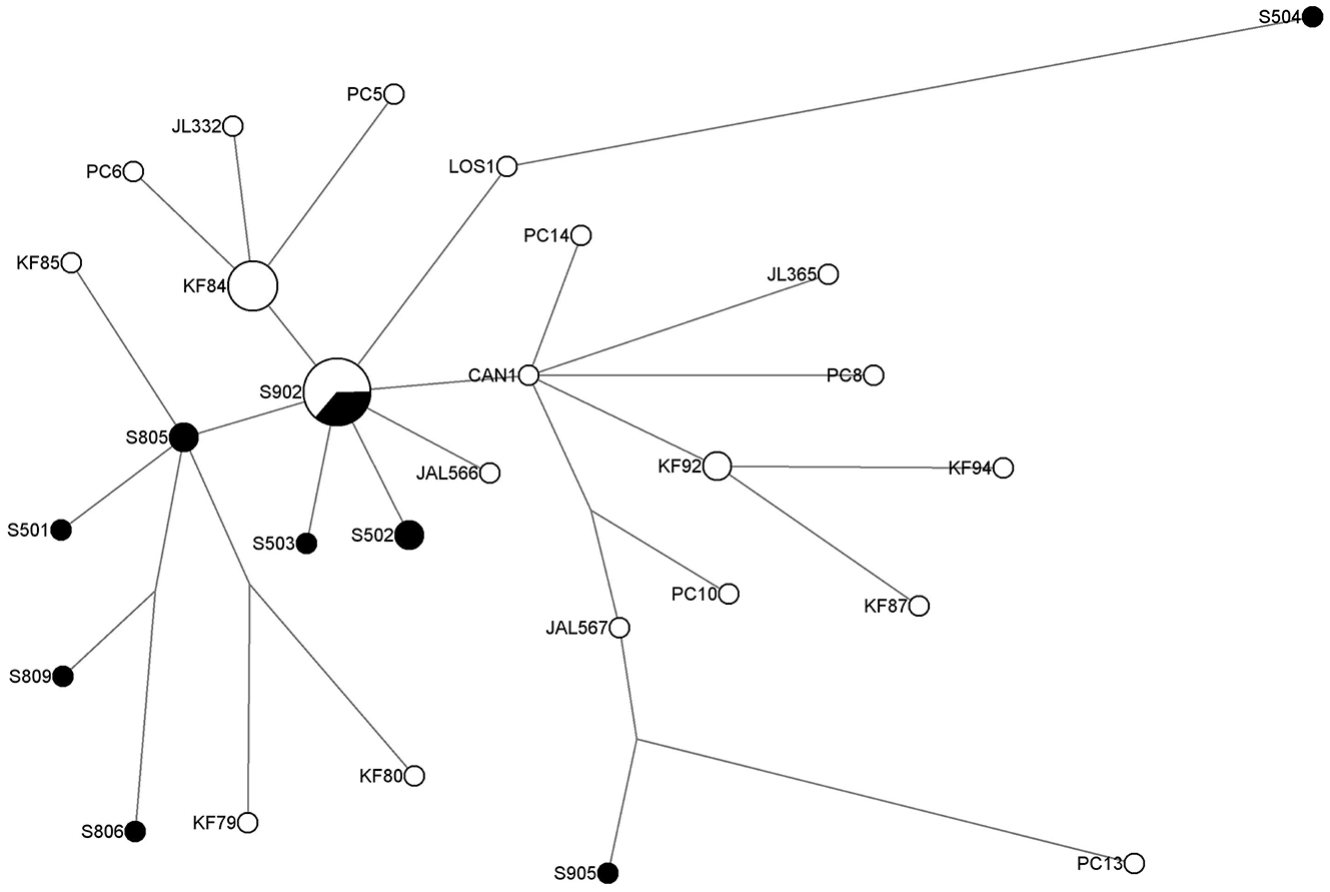
Median-joining network of ancient canid specimens for the mitochondrial DNA control region. Black circles indicate samples from our study (see Table 1 and S6 Table for sample ID). Sequences analyzed in the network span nucleotide positions 15561–15789.

Direct analysis of whole mitogenomes from ancient canid specimens have recently suggested a European origin of domestic dogs based on the phylogenetic arrangement of three canids from Belgium dated to 26,000–36,000 YBP [10], which is in contrast to an Asian or Near East origin based on modern specimens [5,6]. Based on mtDNA control region sequences, the canid specimens from Yana RHS and Ulakhan-Sullar from our study appear as divergent as the ancient European specimens that were used as evidence of a European origin for domestic dogs. Therefore in light of our results, a European origin of domestic dogs may not be conclusive. The median joining network for ancient canid sequences (Fig 2) shows a Yana haplotype (S805) that is one step away from a Zhokhov haplotype (S902) that represents one of the main phylogenetic clades among canids (Clade A). Several ancient canid haplotypes are oriented around the Yana S805 including some of the oldest canid haplotypes reported to date, including the Ulakhan-Sullar specimen (*Canis* cf. *variabilis*, S809) from our study, in addition to ancient canid haplotypes from Belgium dated to 30,000 YBP and 36,000 YBP and a canid haplotype from Kostenki, Russia dated to 22,000 YBP [10]. The ancient canid specimens in this cluster may represent possible progenitors of the domestic dog, as coalescence time for the dog-wolf divergence is thought to have occurred by around 10,000 years ago [4,65] though some have suggested as early as 32,000 years ago [66]. Based on the position of the Yana S805 haplotype, it may potentially represent a direct link from the putative progenitor (including *Canis* cf. *variabilis*) to the domestic dog and modern wolf lineages.

**Fig 3:**
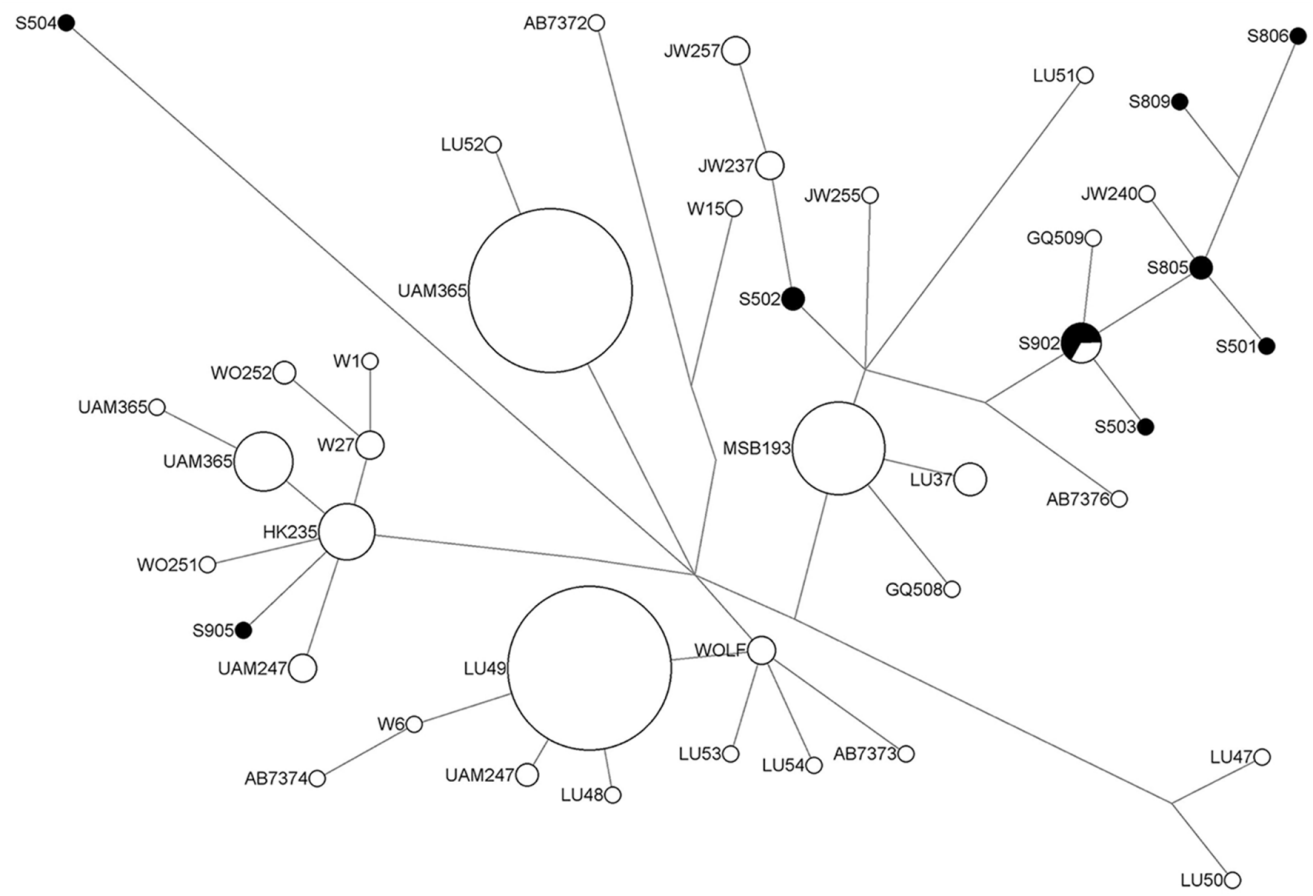
Median-joining network of wolf specimens for the mitochondrial DNA control region. Black circles indicate samples from our study. Sequences analyzed in the network span nucleotide positions 15547–15792. See Table 1 for the sample IDs from this study. Notable haplotypes include GQ509 (GenBank Accession No. GQ376509; Ural mountains of Russia [59]), JW237, 240, 255, 257 (GenBank Accession No. AB480736-AB480742, AB500700; *Canis lupus hodophilax*, Japan [54]), and LU51 (GenBank Accession No. AY812735; New Mexico, USA [57]).

Overall, most of the Siberian canids from our study are phylogenetically clustered with other wolves from Asia and Russia (Fig 3). One branch from the Yana haplotype (S805) consists of a modern wolf haplotype. Interestingly, this haplotype is from a now-extinct Japanese wolf specimen (*Canis lupus hodophilax*) dated from the 14th-18th century [54]. The Japanese wolf is reported to have been fairly widely distributed in the main islands of Japan since the Jomon period (10,000–250 B.C.) until as recent as the early 1900s, but it is unclear where they originate from [54,67]. While it is largely thought that the gray wolf (*Canis lupus*) is directly ancestral to the domestic dog, the phylogenetic relationship between the gray wolf and *Canis* cf. *variabilis* is still a subject of debate. *Canis* cf. *variabilis* is thought to have been widespread in Eurasia until around 300,000 YBP and does not appear to overlap with the earliest occurrence of the morphologically distinctive gray wolf [68,69]. Our study is the first to report DNA results from *Canis* cf. *variabilis* and our results suggest *variabilis* as another possible source of genetic contribution to the domestic dog.

**Fig 4:**
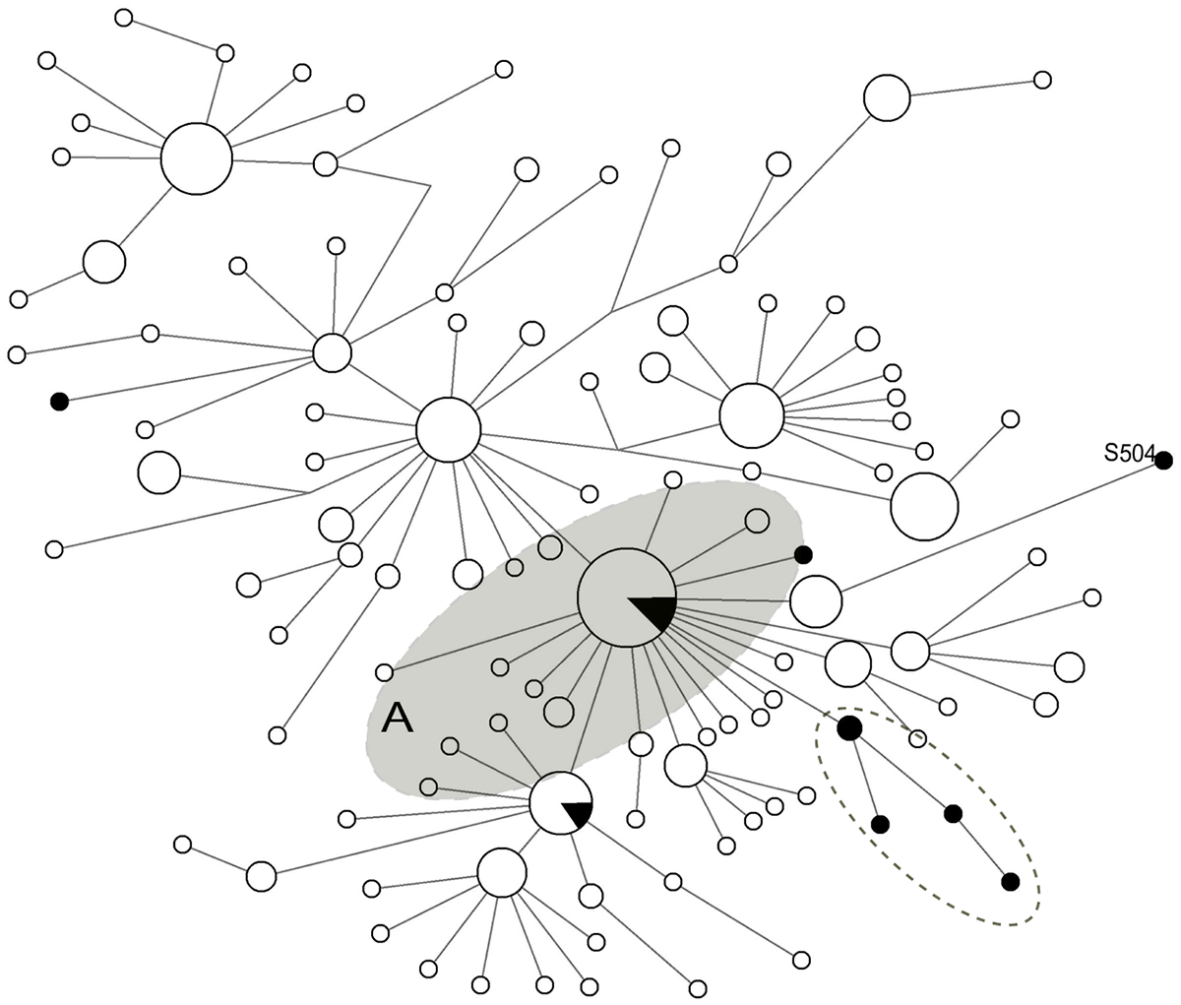
Median-joining network of dog specimens for the mitochondrial DNA control region. Black circles indicate samples from our study. Sequences analyzed in the network span nucleotide positions 15547–15705. Clade A is highlighted in light gray and the Duvanny Yar sample S504 is noted in the network. The bottom-right cluster highlighted with a dashed line circle includes the samples S805, S806, S809, and S501.

One interesting feature in the phylogenetic group of ancient canids surrounding the Yana haplotype (S805) is a Duvanny Yar canid (S504), which is dated to more than 47,000 ^14^C YBP but is separated from the other ancient canid haplotypes and appears to be connected to domestic dogs but not wolves. In both ancient canid and domestic dog median-joining networks (Figs 2 and 4), S504 is connected by dog haplotypes that are a couple of mutations away from Clade A but the wolf network shows S504 genetically divergent from any other haplotypes (Fig 3). This may suggest that the Duvanny Yar canid lineage has a distinct phylogeny from wolves but yet possibly genetically ancestral to domestic dogs. While it is possible that the Duvanny Yar lineage simply did not play a major role in the domestication of modern dog lineages, it may represent a localized genetic source for modern dog lineages.

The phylogenetic network with domestic dog sequences show the cluster oriented around the Yana haplotype (S805) is retained from the wolf network (Fig 4). Both the Zhokhov and Aachim haplotypes situated in Clade A are shared with multiple domestic dogs from a wide geographical distribution [4,53,55]. This major phylogenetic group known as Clade A, along with Clades B, C and D, have been characterized from previously canid studies that includes the majority of domestic dog lineages [53,70]. Both Zhokhov and Aachim haplotypes appear to be genetically indistinguishable from domestic dogs, which may suggest that domestic dogs were in Arctic Siberia by at least 8,000 ^14^C YBP. On the other hand, the Zhokhov and Aachim canids show genetic affinity with Asiatic wolves from Fig 3. In particular, *C*. *lupus chanco* and *C*. *lupus hodophilax* have either shared haplotypes or are separated by one mutation from the Siberian canids, including one Yana canid. While a recent genome-wide analysis of Chinese indigenous dogs showed a closer genetic affinity to domestic dogs [66], another explanation is that the Siberian canids retained a genetic signature from admixture with local breeds through geographical isolation, which has been suggested in other ancient dogs breeds [71].

## Conclusions

Overall, our data suggests a genetic contribution from regional sources of wolves, including possibly *Canis* cf. *variabilis*, to the modern dog lineage and highlights the importance of further exploring the genetic contribution of regional wolf breeds to the domestic dog gene pool. Furthermore, our results are consistent with recent studies suggesting that that dog domestication occurred through a complex process of admixture among diverse breeds integrated with isolation of regional breeds throughout history. Future studies may further illuminate the phylogenetic relationship between the different canid species.

## Supporting Information

S1 Fig Partial mandible of the S809 specimen from Ulakhan Sullar. The specimen was obtained from Layer 2 at Ulakhan-Sullar and described as *Canis* cf. *variabilis*.
(TIF)

S2 Fig Partial mandible of the S504 specimen from Duvanny Yar. Morphologically identified as *Canis lupus*, the specimen was obtained from the Kolyma River downstream.
(TIF)

S1 Table Description of canid remains from Duvanny Yar. Information for the two specimens from Duvanny Yar including their field code, description of remains, location, and details of radiocarbon dating.
(DOCX)

S2 Table Description of canid specimens from Yana RHS. Information for the four specimens from Yana RHS including their field code, description of remains, location, and details of radiocarbon dating.
(DOCX)

S3 Table Description of canid specimens from Zhokhov. Information for the four specimens from Zhokhov including their field code, description of remains, location, and details of radiocarbon dating.
(DOCX)

S4 Table Description of canid specimens from the Aachim Lighthouse. Information for the two specimens from Aachim including their field code, description of remains, location, and details of radiocarbon dating.
(DOCX)

S5 Table Mitochondrial DNA primer sequences used in this study. Three primer pairs were designed for the study and one primer pair was used from a previous study [64].
(DOCX)

S6 Table Samples used in median-joining network of ancient canid specimens. The ID codes in Fig 2 correspond to the indicated GenBank Accession numbers and reference.
(DOCX)
