# Supplementary figures and images for: Ancient DNA Analysis of the Oldest Canid Species from the Siberian Arctic and Genetic Contribution to the Domestic Dog
Source: PLoS One. 2015 May 27;10(5):e0125759. doi: 10.1371/journal.pone.0125759 (PMC4446326; doi:10.1371/journal.pone.0125759)

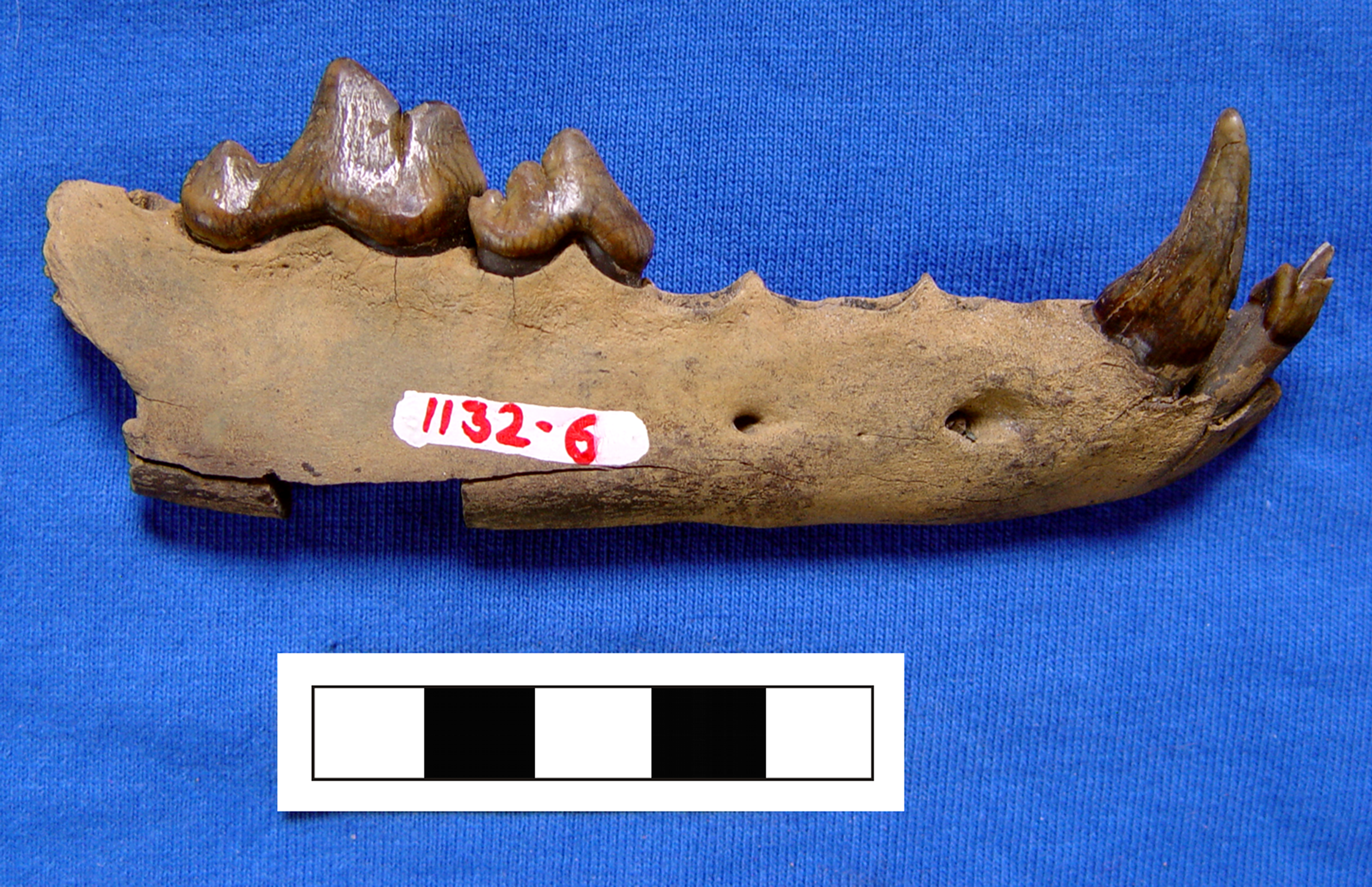

Supplement: S1 Fig — The specimen was obtained from Layer 2 at Ulakhan-Sullar and described as Canis cf. variabilis. (TIF) [file pone.0125759.s001.tif]

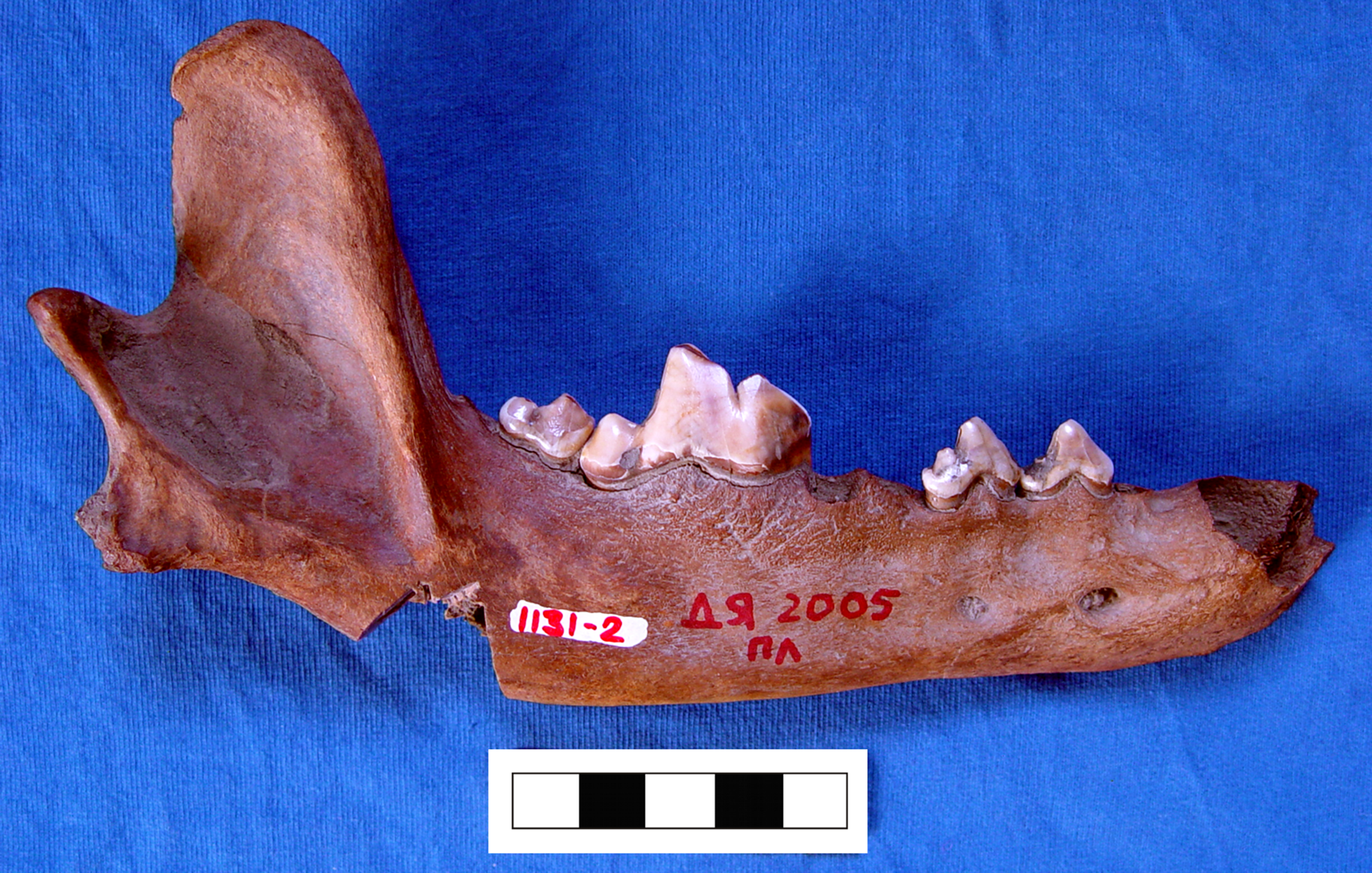

Supplement: S2 Fig — Morphologically identified as Canis lupus, the specimen was obtained from the Kolyma River downstream. (TIF) [file pone.0125759.s002.tif]
