# Supplementary material for: Ancient DNA Analysis of the Oldest Canid Species from the Siberian Arctic and Genetic Contribution to the Domestic Dog
Source: PLoS One. 2015 May 27;10(5):e0125759. doi: 10.1371/journal.pone.0125759 (PMC4446326; doi:10.1371/journal.pone.0125759)
Supplement: S1 Table — Information for the two specimens from Duvanny Yar including their field code, description of remains, location, and details of radiocarbon dating. (DOCX) [file pone.0125759.s003.docx]

S1 Table. Description of canid remains from Duvanny Yar

| Sample code | Field code | Description | Location | Sample code for Beta Analytic Inc. | Individual ^14^C AMS date |
| --- | --- | --- | --- | --- | --- |
| S503 | #4 | *Canis lupus:* lower canine | Duvanny Yar exposure, Kolyma River downstream | MA-2265 | >47000  Beta-231445 |
| S504 | DYAR-dop | *Canis lupus:* mandible | Kolyma River downstream | MA-2266 | >47000  Beta-231446 |
