# Supplementary material for: Ancient DNA Analysis of the Oldest Canid Species from the Siberian Arctic and Genetic Contribution to the Domestic Dog
Source: PLoS One. 2015 May 27;10(5):e0125759. doi: 10.1371/journal.pone.0125759 (PMC4446326; doi:10.1371/journal.pone.0125759)
Supplement: S2 Table — Information for the four specimens from Yana RHS including their field code, description of remains, location, and details of radiocarbon dating. (DOCX) [file pone.0125759.s004.docx]

S2 Table. Description of canid specimens from Yana RHS

| Sample code | Field code | Description | Location | Sample code for Beta Analytic Inc. | Individual ^14^C AMS date |
| --- | --- | --- | --- | --- | --- |
| S501 | Y06 NP 19251 | *Canis lupus*: upper left canine teeth of young animal, with no visible worn traces | Unit N26 | MA-2263 | 28520+/-240 Beta-231443 |
| S601 | Y06 NP 21016 | *Canis lupus*: low left canine teeth of adult animal, seriously worn | Unit M26 | MA-2267 | 27840+/220 Beta-231447 |
| S805 | Y06 NP 18994 | *Canis lupus*: low left canine teeth of adult animal, medium worn (root portion sampled) | Unit M24 |  | n/a |
| S806 | Y06 NP 17153 | *Canis lupus*: low left canine teeth of adult/old(?) animal, seriously worn | Unit L24 |  | n/a |
