# Supplementary material for: Ancient DNA Analysis of the Oldest Canid Species from the Siberian Arctic and Genetic Contribution to the Domestic Dog
Source: PLoS One. 2015 May 27;10(5):e0125759. doi: 10.1371/journal.pone.0125759 (PMC4446326; doi:10.1371/journal.pone.0125759)
Supplement: S3 Table — Information for the four specimens from Zhokhov including their field code, description of remains, location, and details of radiocarbon dating. (DOCX) [file pone.0125759.s005.docx]

S3 Table. Description of canid specimens from Zhokhov

| Sample code | Field code | Description | Location | Sample code for Beta Analytic Inc. | Individual ^14^C AMS date |
| --- | --- | --- | --- | --- | --- |
| S602 | Zhokh2003-85/P2+M2, | *Canis sp.:* upper left premolar + upper right molar, both seriously worn | Zhokhov site, cultural layer, unit BC13 | MA-2268 | 8710+/-50  Beta-231448 |
| S902 | Zhokh2004-19 | *Canis sp***.:** low left canine teeth of young animal, with almost no worn | Zhokhov site, cultural layer, unit Nn220 |  | n/a |
| S904 | Zhokh2004-18 | *Canis sp***.:** low left canine teeth, medium worn | Zhokhov site, cultural layer, unit Nn190 |  | n/a |
| S903 | Zhokh2004-113 | *Canis sp***.:** low left canine teeth of very young animal, no worn | Zhokhov site, cultural layer, unit Fff260 |  | n/a |
