# Supplementary material for: Ancient DNA Analysis of the Oldest Canid Species from the Siberian Arctic and Genetic Contribution to the Domestic Dog
Source: PLoS One. 2015 May 27;10(5):e0125759. doi: 10.1371/journal.pone.0125759 (PMC4446326; doi:10.1371/journal.pone.0125759)
Supplement: S4 Table — Information for the two specimens from Aachim including their field code, description of remains, location, and details of radiocarbon dating. (DOCX) [file pone.0125759.s006.docx]

S4 Table. Description of canid specimens from the Aachim Lighthouse

| Sample code | Field code | Description | Sample code for Beta Analytic Inc. | Individual ^14^C AMS date |
| --- | --- | --- | --- | --- |
| S603 | Aachim1999-AM1 | *Canis sp***.:** low right canine, medium worn | MA-2269 | 1760+/-40  Beta-231449 |
| S502 | Aachim1999-AM2/Р3 | *Canis sp.:* low right third premolar, slightly worn + a mandible fragment | MA-2264 | 1740+/40  Beta-231444 |
