# Supplementary material for: Ancient DNA Analysis of the Oldest Canid Species from the Siberian Arctic and Genetic Contribution to the Domestic Dog
Source: PLoS One. 2015 May 27;10(5):e0125759. doi: 10.1371/journal.pone.0125759 (PMC4446326; doi:10.1371/journal.pone.0125759)
Supplement: S5 Table — Three primer pairs were designed for the study and one primer pair was used from a previous study [64]. (DOCX) [file pone.0125759.s007.docx]

S5 Table. Mitochondrial DNA primer sequences used in this study

| Primer | Sequence (5' - 3') | Reference |
| --- | --- | --- |
| 15441F | TCTTCTTAAACTATTCCCTG | this study |
| 15583R | ACATGCTTATATGCATGGG | this study |
| 15424F | GCACCCAAAGCTGAAATTC | this study |
| 15578R | CTTATATGCATGGGGCAAA | this study |
| 15541F | CGTCGTGCATTAATGGTTTGC | [1] |
| 15692R | CATGGTGATTAAGCCCTTATTGGA | [1] |
| 15657F | ATGCATATCACTTAGTCCA | this study |
| 15837R | AGTAGGATTGGAGTAAATA | this study |

Reference:

1. Malmström H, Stora J, Dalen L, Holmlund G, Gotherstrom A (2005) Extensive human DNA contamination in extracts from ancient dog bones and teeth. Molecular Biology and Evolution 22: 2040-2047.
