# Supplementary material for: Ancient DNA Analysis of the Oldest Canid Species from the Siberian Arctic and Genetic Contribution to the Domestic Dog
Source: PLoS One. 2015 May 27;10(5):e0125759. doi: 10.1371/journal.pone.0125759 (PMC4446326; doi:10.1371/journal.pone.0125759)
Supplement: S6 Table — The ID codes in Fig 2 correspond to the indicated GenBank Accession numbers and reference. (DOCX) [file pone.0125759.s008.docx]

S6 Table. Samples used in median-joining network of ancient canid specimens

| ID | GenBank Accession No. | Reference |
| --- | --- | --- |
| PC5, 8, 10, 13, 14 | AY163884, AY163886-87, AY163889-90 | [1] |
| LOS1 | n.a. | [2] |
| JL332, 365 | AY163880, AY163883 | [1] |
| KF79, 80, 84, 85, 87, 92, 94 | KF661079-80, KF661084-85, KF661087, KF661092, KF661094 | [3] |
| JAL566, 567 | KC776175, KC776179 | [4] |
| CAN1 | JX173682 | [5] |

References:

1. Leonard JA, Wayne RK, Wheeler J, Valadez R, Guillen S, Vila C (2002) Ancient DNA evidence for Old World origin of New World dogs. Science 298: 1613-1616.

2. Losey RJ, Bazaliiskii VI, Garvie-Lok S, Germonpré M, Leonard JA, Allen AL, et al. (2011) Canids as persons: Early Neolithic dog and wolf burials, Cis-Baikal, Siberia. Journal of Anthropological Archaeology 30: 174-189.

3. Thalmann O, Shapiro B, Cui P, Schuenemann VJ, Sawyer SK, Greenfield DL, et al. (2013) Complete mitochondrial genomes of ancient canids suggest a European origin of domestic dogs. Science 342: 871-874.

4. Losey RJ, Garvie-Lok S, Leonard JA, Katzenberg MA, Germonpré M, Nomokonova T, et al. (2013) Burying dogs in ancient Cis-Baikal, Siberia: temporal trends and relationships with human diet and subsistence practices. PLoS ONE 8: e63740.

5. Druzhkova AS, Thalmann O, Trifonov VA, Leonard JA, Vorobieva NV, et al. (2013) Ancient DNA analysis affirms the canid from Altai as a primitive dog. PLoS ONE 8: e57754.
